# Reproducibility and repeatability of quantitative T2 and T2* mapping of osteosarcomas in a mouse model

**DOI:** 10.1186/s41747-024-00467-9

**Published:** 2024-06-14

**Authors:** Raheleh Roudi, Laura J. Pisani, Fabrizio Pisani, Tie Liang, Heike E. Daldrup-Link

**Affiliations:** 1 Department of Radiology, Molecular Imaging Program at Stanford, Stanford University, Stanford, CA 94305 USA; 2 Department of Pediatrics, Hematology/Oncology, Stanford University School of Medicine, Stanford, CA USA

**Keywords:** Magnetic iron oxide nanoparticles, Magnetic resonance imaging, Mice (inbred BALB/c), Osteosarcoma, Tumor-associated macrophages

## Abstract

**Background:**

New immunotherapies activate tumor-associated macrophages (TAMs) in the osteosarcoma microenvironment. Iron oxide nanoparticles (IONPs) are phagocytosed by TAMs and, therefore, enable TAM detection on T2*- and T2-weighted magnetic resonance images. We assessed the repeatability and reproducibility of T2*- and T2-mapping of osteosarcomas in a mouse model.

**Methods:**

Fifteen BALB/c mice bearing-murine osteosarcomas underwent magnetic resonance imaging (MRI) on 3-T and 7-T scanners before and after intravenous IONP infusion, using T2*-weighted multi-gradient-echo, T2-weighted fast spin-echo, and T2-weighted multi-echo sequences. Each sequence was repeated twice. Tumor T2 and T2* relaxation times were measured twice by two independent investigators. Repeatability and reproducibility of measurements were assessed.

**Results:**

We found excellent agreement between duplicate acquisitions for both T2* and T2 measurements at either magnetic field strength, by the same individual (repeatability), and between individuals (reproducibility). The repeatability concordance correlation coefficient (CCC) for T2* values were 0.99 (coefficients of variation (CoV) 4.43%) for reader 1 and 0.98 (CoV 5.82%) for reader 2. The reproducibility of T2* values between the two readers was 0.99 (CoV 3.32%) for the first acquisitions and 0.99 (CoV 6.30%) for the second acquisitions. Regarding T2 values, the repeatability of CCC was similar for both readers, 0.98 (CoV 3.64% for reader 1 and 4.45% for reader 2). The CCC of the reproducibility of T2 was 0.99 (CoV 3.1%) for the first acquisition and 0.98 (CoV 4.38%) for the second acquisition.

**Conclusions:**

Our results demonstrated high repeatability and reproducibility of quantitative T2* and T2 mapping for monitoring the presence of TAMs in osteosarcomas.

**Relevance statement:**

T2* and T2 measurements of osteosarcomas on IONP-enhanced MRI could allow identifying patients who may benefit from TAM-modulating immunotherapies and for monitoring treatment response. The technique described here could be also applied across a wide range of other solid tumors.

**Key points:**

• Optimal integration of TAM-modulating immunotherapies with conventional chemotherapy remains poorly elucidated.

• We found high repeatability of T2* and T2 measurements of osteosarcomas in a mouse model, both with and without IONPs contrast, at 3-T and 7-T MRI field strengths.

• T2 and T2* mapping may be used to determine response to macrophage-modulating cancer immunotherapies.

**Graphical Abstract:**

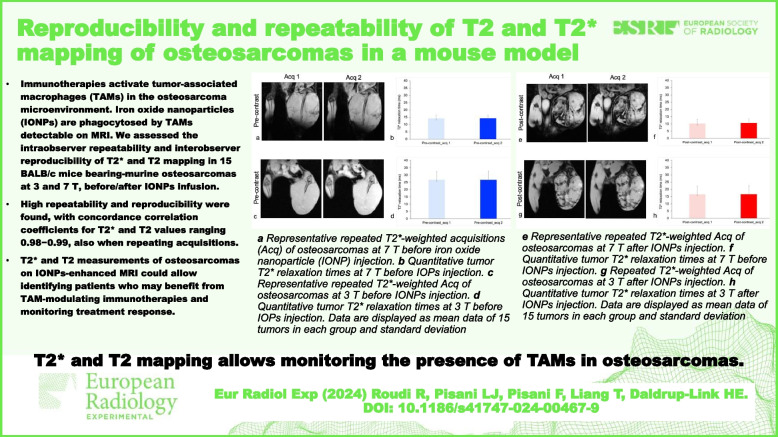

**Supplementary Information:**

The online version contains supplementary material available at 10.1186/s41747-024-00467-9.

## Background

Osteosarcoma is the most common bone tumor in children and adolescents. The 5-year survival rate of metastatic osteosarcoma is 30% [1]. Therefore, new treatment options are being investigated. New immunotherapies that activate tumor-associated macrophages (TAMs) in the osteosarcoma microenvironment have demonstrated promising results [2]. Since the tumor does not change in size in response to immunotherapy, at least not in the immediate post-treatment phase, an imaging technology that can visualize TAM activation would greatly help to identify responders to these new therapies. Intravenously injected iron oxide nanoparticles (IONPs) are phagocytosed by TAMs and shorten T2* and T2 relaxation times of osteosarcomas on magnetic resonance imaging (MRI) [3–5]. A hypointense contrast enhancement, as quantified by decreasing tumor T2* and T2 relaxation times, correlated with the quantity of macrophages within the tumor on histology [5, 6]. IONP-enhanced MRI can therefore help to detect TAMs in osteosarcomas, using T2* and T2 mapping techniques. However, the reproducibility and repeatability of these tumor T2* and T2 measurements have not been investigated in osteosarcomas.

The repeatability and reproducibility of T2* and T2 measurements for measurements of the iron content in the liver and heart muscle have been studied [7–9]. Several research groups demonstrated excellent to good agreement in intra-observer and inter-observer reproducibility in the T2* measurement of liver tissue [10–12]. Similarly, the T2* measurement of myocardial tissue has shown consistent reproducibility in different studies [8, 11, 13]. However, the accumulation of endogenous iron in the liver and heart muscle in the setting of hemosiderosis is typically homogenous, whereas the accumulation of IONPs in solid tumors is typically heterogeneous.

Relatively few publications report the reproducibility of T2* or T2 measurement in tumors [14, 15]. The reproducibility of R2* (R2* = 1/T2*) measurements of prostate adenocarcinoma was reported to be 64.6% [14] and the reproducibility of T2* measurements of liver metastasis in patients with colorectal cancer was reported to be 55.4% for 84th percentiles [15]. While we recognize that tumor tissue is more heterogeneous than that of most normal organs, we hypothesized that standardized approaches should yield significant agreements between repeated acquisitions and repeated measurements of tumor T2* and T2 values.

The purpose of our study was to assess the repeatability and reproducibility of T2* and T2 mapping as imaging biomarkers of TAM activation in a mouse osteosarcoma model.

## Methods

### Cell line culture

The murine osteosarcoma K7M2 (CRL-2836™) cell line was obtained from the American Type Culture Collection and cultured in Dulbecco’s Modified Eagle’s Medium supplemented with 10% fetal bovine serum, 100 mg/mL streptomycin, 100 U/mL penicillin and 2 mM L-glutamine. All cell culture reagents were provided by Gibco, Invitrogen, Carlsbad, CA USA. Cells were maintained at 37 °C in a humidified incubator infused with 5% CO2.

### Osteosarcoma mouse model

**Fig. 1:**
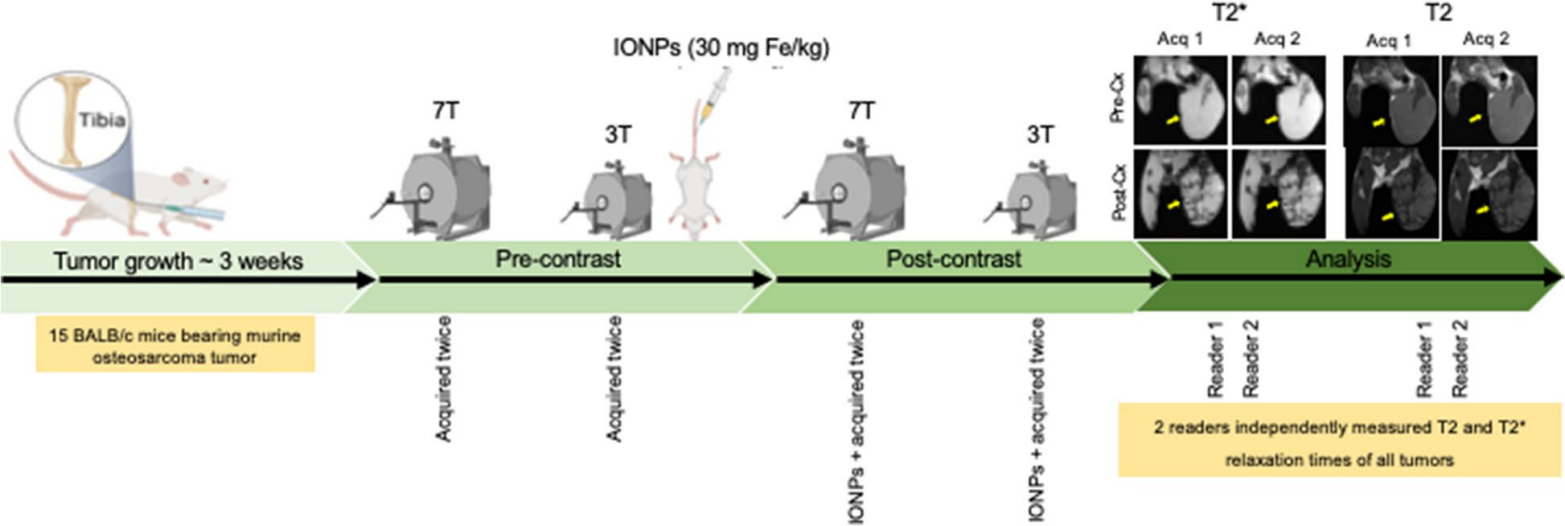
Experimental design: K7M2 murine osteosarcoma cells were implanted into the proximal right tibia of BALB/c mice. After 3 weeks, tumor-bearing mice underwent magnetic resonance imaging (MRI) on 7-T and 3-T scanners. Multiecho T2- and T2*-weighted sequences were acquired twice (acquisition 1 [Acq 1] and acquisition 2 [Acq 2]). Immediately after pre-contrast scanning (pre-Cx), iron oxide nanoparticles (IONPs) were injected through the tail vein at a dose of 30 Fe mg/kg. Twenty-four hours later, animals underwent post-contrast scanning (post-Cx) on both 7-T and 3-T scanners. The yellow arrows indicate tumors on T2-weighted images. A region of interest was used to delineate the tumors on these images

All experimental procedures involving mice were approved by the Stanford University Administrative Panel on Laboratory Animal Care (Protocol 24,965). Fifteen female 6- to 8-week-old BALB/c mice (000651, Jackson Laboratory, Bar Harbor, ME, USA) were included in the experiment (Fig. 1). The mice were anesthetized via inhalation of 1.5−2.0% isoflurane in oxygen, and then 1 × 10^5^ K7M2 tumor cells suspended in phosphate-buffered saline were implanted into the right proximal tibial metaphysis. Tumors with intraosseous and extraosseous soft tissue components, resembling the growth pattern of human tumors, typically grew to a size of 1 cm within 3 weeks after inoculation.

### MRI scans

When the tumor had reached a size of 1 cm, tumor-bearing mice underwent MRI on 7-T and 3-T scanners (both Bruker Corporation, Billerica, MA, USA) with the parameters shown in Table 1. T2-weighted fast spin-echo images were acquired for anatomic reference and region of interest definition. Multislice and multiecho gradient-echo and spin-echo sequences were acquired for T2*- and T2-map calculations. Each mapping sequence was acquired twice. Technical parameters of both sequences at 3 T and 7 T are shown in Table 1. Immediately after MRI scanning, each mouse received a single intravenous injection of IONPs (Ferumoxytol; AMAG Pharmaceuticals, Inc., Waltham, MA, USA) at a dose of 30 Fe mg/kg (Fig. 1). Twenty-four hours after the IONP injection, animals underwent MRI on both 3-T and 7-T scanners as described above.

**Table 1:** Acquisition parameters of multislice sequences

Magnetic field strength	3 T		7 T	
Parameter measured/Sequence	T2*	T2	T2*	T2
	Gradient-echo	Spin-echo	Gradient-echo	Spin-echo
Repetition time (ms)	800	1,800	800	2,200
Minimum echo time (ms)	3	12	3.5	7.5
Echo train length	12	10	10	13
Echo spacing (ms)	4	12	5	7.5
Flip angle (degree)	60	180	50	180
Number of excitations	4	2	2	1
Field of view (mm)	60	60	25.6	25
Sampling matrix (pixels)	160 × 160	160 × 160	256 × 256	192 × 192
Slice thickness (mm)	1	1	1	0.8

### T2* and T2 mapping

T2* and T2-maps were calculated based on the multi-gradient-echo and multi-slice multi-echo acquisitions, respectively. A mono-exponential decay model with a constant offset was fit to the signal in the input images, *S*(*t*), and the time of each echo, *t*, according to:

$$S\left(t\right)=A+S_o^\ast\text{exp}\left(-t/T\right)$$

where the parameters calculated by the model are *A* = absolute bias, *S*_*o*_ = initial signal intensity, and *T* = either T2* or T2. Those parameters are evaluated and mapped in Paravision 360 (3-T) and 7.1 (7-T) (Bruker Corp, Billerica MA, USA).

### Parametric data analysis

MRI anatomic and parametric data were exported for analysis to Osirix version 8.0 (Pixmeo SARL, Bernex, Switzerland). Two trained researchers (R.R. and F.P.) independently measured T2* and T2 relaxation times of all tumors on pre-contrast and post-contrast acquisitions at both field strengths. These eight measurements were performed twice by each researcher. The standardized analysis procedure in Osirix was as follows:

1. Outline the entire tumor by manually drawing regions of interest circumscribing the tumor on each slice of the T2-weighted anatomic images;
2. Add a mask to exclude pixels below 7% (7 T) and 30% (3 T) of the signal of normal muscle (*i.e.*, the mean signal value in the calf muscle of the contralateral leg) on the minimum TE images; and
3. Record the mean T2* and T2 relaxation times of the tumor entire tumor volume.

### Statistical analysis

Descriptive data of tumor T2* and T2 relaxation times are shown as mean data ± standard deviation. Shapiro–Wilk and Shapiro-Francia tests were conducted to prove normality distribution of the data. A linear regression model was used to correlate T2* or T2 with field strength. Statistical significance was considered for *p*-values < 0.05.

The agreement between T2* and T2 measurements on first and second acquisitions by each reader (intraobserver) refers to the repeatability, and agreement between the two readers (inter-observer) refers to the reproducibility. Repeatability and reproducibility were examined using concordance correlation coefficient (CCC) and Bland–Altman analysis and the mean difference is presented with 95% limits of agreement (LOA) [16, 17]. In addition, we used coefficients of variation (CoV) to evaluate the distribution of the data relative to their average [18]. The CoV (%) is defined as the standard deviation of the means divided by the average of means. The CoV was assessed as poor (CoV > 20%), moderate (10% < CoV ≤ 20%), good (5% < CoV ≤ 10%), or excellent (CoV ≤ 5%) [19].

CoV is a measure of precision and provides an indication of how spread out the data are. However, CoV does not address bias, which refers to systematic error in measurements. CCC assesses the agreement between two sets of measurements, considering not only precision but also whether they have a bias. A CCC of 1 indicates perfect agreement, 0 indicates no agreement beyond chance, and -1 indicates perfect disagreement. Therefore, CoV is valuable for assessing precision, whereas CCC provides a more comprehensive assessment of accuracy by considering both precision and bias [16].

## Results

### T2*- and T2-weighted MRI

**Fig. 2:**
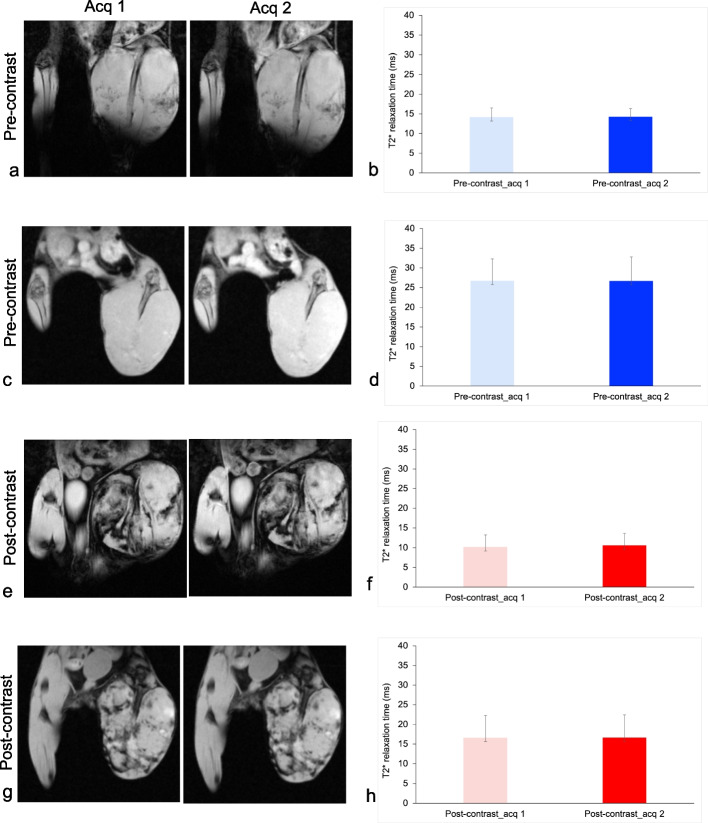
Reproducibility of T2* measurements. **a** Representative repeated T2*-weighted acquisitions (Acq) of osteosarcomas at 7 T before iron oxide nanoparticle (IONP) injection. **b** Quantitative tumor T2* relaxation times at 7 T before IONP injection. **c** Representative repeated T2*-weighted Acq of osteosarcomas at 3 T before IONP injection. **d** Quantitative tumor T2* relaxation times at 3 T before IONP injection. **e** Representative repeated T2*-weighted Acq of osteosarcomas at 7 T after IONP injection. **f** Quantitative tumor T2* relaxation times at 7 T before IONP injection. **g** Representative repeated T2*-weighted Acq of osteosarcomas at 3 T after IONP injection. **h** Quantitative tumor T2* relaxation times at 3 T after IONP injection. All quantitative data represent the mean data of 15 tumors in each group and standard deviations

**Fig. 3:**
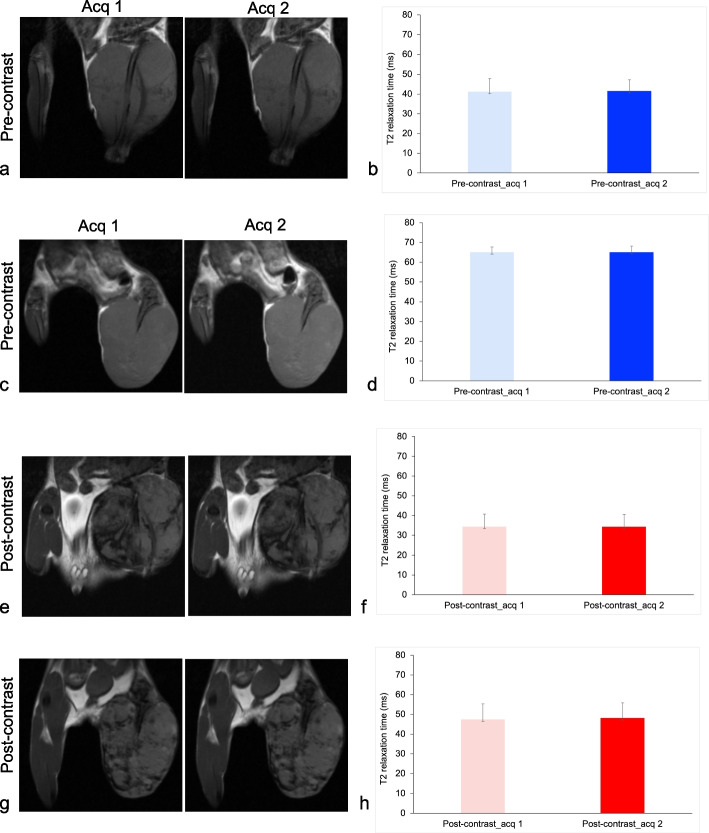
Reproducibility of T2 measurements. **a** Representative repeated T2-weighted acquisitions (Acq) of osteosarcomas at 7 T before iron oxide nanoparticle (IONP) injection. **b** Quantitative tumor T2 relaxation times at 7 T before IONP injection. **c** Representative repeated T2-weighted Acq of osteosarcomas at 3 T before IONPs injection. **d** Quantitative tumor T2 relaxation times at 3 T before IONP injection. **e** Representative repeated T2-weighted Acq of osteosarcomas at 7 T after IONP injection. **f** Quantitative tumor T2 relaxation times at 7 T after IONP injection. **g** Representative repeated T2-weighted acquisitions of osteosarcomas at 3 T IONP injection. **h** Quantitative tumor T2 relaxation times at 3 T after IONP injection. All quantitative data represent the mean data of 15 tumors in each group and standard deviations

Pre-contrast MRI of the 15 female BALB/c mice confirmed osteosarcoma tumor growth. These images showed the development of a primary tumor in the proximal tibia, with an intraosseous tumor component, cortical disruption, and extraosseous soft tissue component, similar to the growth pattern in human patients (Figs. 2 and 3). Our analysis demonstrated normal distribution of T2* relaxation times for both first and second acquisitions as well as reader 1 and reader 2. In addition, T2* relaxation times showed normal distribution in terms of acquisitions and readers.

Pre-contrast T2* relaxation times of osteosarcomas were significantly shorter at 7 T (first acquisition, 14.2 ± 2.35 ms; second acquisition, 14.28 ± 2.08 ms; Fig. 2a, b) than at 3 T (first acquisition, 26.75 ± 5.56 ms; second acquisition, 26.71 ± 6.09 ms; *p* < 0.001; Fig. 2c, d). At 24 h after IONP infusion, all tumors demonstrated significant shortening of T2* relaxation times at 7 T (first acquisition, 10.21 ± 3 ms; second acquisition, 10.64 ± 2.97 ms; Fig. 2e, f) and at 3 T (first acquisition,16.63 ± 5.67 ms; second acquisition, 16.70 ± 5.73 ms, *p* < 0.001, Fig. 2g, h and Supplemental Fig. S1a).

Similarly, pre-contrast T2 relaxation times of osteosarcomas were significantly shorter at 7 T (first acquisition, 41.24 ± 6.58 ms; second acquisition, 41.60 ± 5.68 ms, Fig. 3a, b) than at 3 T (first acquisition 65.06 ± 2.64 ms; second acquisition, 65.11 ± 3.01 ms; *p* < 0.001; Fig. 3c, d). At 24 h after IONP infusion, all tumors demonstrated significant shortening of T2 relaxation times at 7 T (first acquisition, 34.44 ± 6.26 ms; second acquisition, 34.42 ± 6.13 ms; Fig. 3e, f) and at 3 T (first acquisition, 47.46 ± 7.89 ms; second acquisition, 48.18 ± 7.70 ms; *p* < 0.001, Fig. 3g, h and Supplemental Fig. S1b).

### Repeatability of tumor T2* and T2 relaxation times

**Fig. 4:**
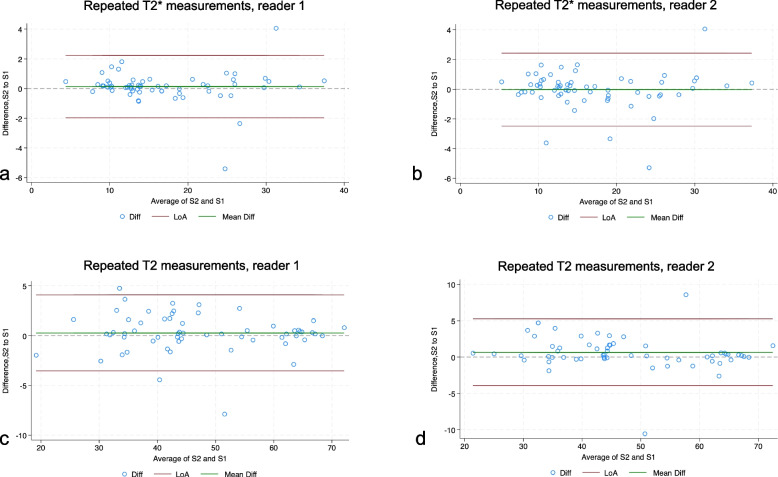
Repeatability of tumor T2* and T2 measurements. Bland–Altman plots of the difference between the first and second T2* measurements of the same tumor by reader 1 (**a**), and reader 2 (**b**). Bland–Altman plots of the difference between the first and second T2 measurements of the same tumor by reader 1 (**c**), and reader 2 (**d**). The green line indicates the mean difference, and the red lines indicate the 95% limits of agreement (LoA)

The intra-observer analyses of repeated T2* measurements demonstrated a strong agreement between the first and second T2* sequences for both readers using CCC. The intraobserver CCC for T2* values were 0.99 (95% CI 0.98−0.99, Fig. 4a) for reader 1 and 0.98 (95% CI (0.97−0.99, Fig. 4b) for reader 2. Concerning T2 measurements, an excellent intraobserver agreement was noted for readers 1 and 2. As depicted in Fig. 4c, d, intraobserver CCC was 0.98 (95% CI 0.98−0.99) and 0.98 (95% CI 0.97−0.98) for readers 1 and 2, respectively.

### Reproducibility of tumor T2* and T2 relaxation times

**Fig. 5:**
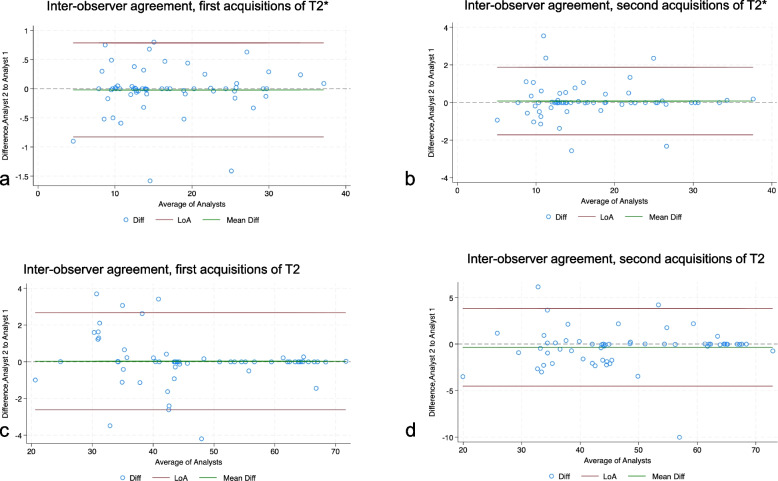
Reproducibility of tumor T2* and T2 measurements. Bland–Altman plots of the difference between the first T2* measurements of the same tumor between two readers (**a**), and Bland–Altman plots of the difference between the second T2* measurements of the same tumor between two readers (**b**). Bland–Altman plots of the difference between the first T2 measurements of the same tumor between two readers (**c**), and Bland–Altman plots of the difference between the second T2 measurements of the same tumor between two readers (**d**). The green line indicates the mean difference, and the red lines indicate the 95% limits of agreement (LoA)

We found an inter-observer variability of less than 5% for repeated measurements of T2* and T2 values. The inter-observer agreement of repeated T2* measurements by two readers was 0.99 (95% CI 0.99−0.99) for the first acquisitions (Fig. 5a) and 0.99 (95% CI 0.98−0.99) for the second acquisitions (Fig. 5b). The Bland–Altman plot also showed a strong inter-observer agreement of T2 values for the first (0.99, 95% CI 0.99−0.99), Fig. 5c) and second acquisitions (0.98, 95% CI 0.97−0.99, Fig. 5d).

### Coefficients of variation

As summarized in Table 2, the CoV for the first acquisition of T2* values was 3.32% indicating low variability. The CoV for the second acquisition obtained from T2*-mapping was 6.30%. Excellent agreement of 4.43% was seen in the T2* measurements by reader 1, and CoV was 5.82% for reader 2 for T2* values. The T2 estimates obtained from the first and second acquisitions also showed excellent agreement (3.1% and 4.38%, respectively). Both readers had an excellent agreement in T2-map measurements (Reader 1, 3.64%; Reader 2, 4.45%).

**Table 2:** Coefficients of variation (CoV) of T2* and T2 relaxation time measurement in repeated acquisitions by two readers

Parameters, acquisitions, readers		CoV (%)
T2* relaxation time (ms)	First acquisition	3.32
	Second acquisition	6.30
	Reader 1	4.43
	Reader 2	5.82
T2 relaxation time (ms)	First acquisition	3.1
	Second acquisition	4.38
	Reader 1	3.64
	Reader 2	4.45

## Discussion

Our research has established the repeatability and reproducibility of T2* and T2 quantification in an animal model of osteosarcoma, before and after the administration of IONP contrast, and at 3-T and 7-T MRI field strengths. Furthermore, our results demonstrate minimal intra- and inter-observer variability in T2* and T2 measurements in our osteosarcoma mouse model.

Quantitative imaging has progressively gained importance in both preclinical research and clinical imaging applications. Our data showed in accordance with the literature that post-contrast T2* and T2 values are shorter than their pre-contrast counterparts at both field strengths [10]. As anticipated, T2* and T2 values at 3 T were higher than at 7 T.

Our findings regarding the low variability of repeated T2* and T2 measurements at both field strengths align with previous studies across various diseases and organs [20–24]. For instance, Li et al. demonstrated the excellent reproducibility of T2 measurements at 3 T in cervical cancer [20], while Vietti Violi et al. exhibited strong intra- and inter-observer agreement in measuring T2 at 3T in different anatomical locations of the pancreas [21]. Ge et al. noted a shorter T2 signal decay time in patients with malignant lymph nodes, underlining the diagnostic potential of T2, with high intra- and inter-observer agreement [22]. Similarly, analysis of the lumbar spine in healthy volunteers indicated excellent repeatability in T2 measurements [23]. All of these previous studies focused on the repeatability and reproducibility of T2 and T2* measurements on unenhanced MRI images, whereas our study investigated the repeatability and reproducibility of T2 and T2* measurements after IONP administration.

We found an inter-observer variability of less than 5% for T2* and T2 measurements of osteosarcomas, which is consistent with the reported inter-observer variability in other tissues and other tumors.

For T2* measurements of iron overload in the liver, Positano et al. reported an intra- and inter-observer variability of 3.7% and 5.6%, respectively [10], Kirk reported an inter-observer variability of 4.4% [11] and Meloni et al. reported an intra- and inter-observer variability of 4% and 6.9%, respectively [12]. For T2* measurements of iron overload in the heart, Anderson et al. reported an inter-study variability of 5% [8], Westwood et al. reported a variability of 3.5% and 2.4% at two different centers for T2* [13] and Kirk et al. reported an inter-study and inter-observer variability of 5.9% and 5.4%, respectively [11]. Panek et al. found that T2* analysis is a sensitive approach for the clinical detection of oxygenation levels in head and neck squamous cell carcinoma at 3T [25].

In conclusion, we found significant repeatability and reproducibility of T2* and T2 measurements of osteosarcomas in a mouse model, both with and without IONP contrast, at 3T and 7T MRI field strengths. Furthermore, we have demonstrated strong intra- and inter-observer consistency of these measurements.

### Supplementary Information

**Additional file 1:** **Supplementary Fig. S1.** Tumor T2* and T2 relaxation time measurements before and after iron oxide nanoparticle (IONP) injection: (a) Tumor T2* relaxation time at 3 T and 7 T before (pre) and after (post) IONP injection. (b) T2 relaxation time (ms) at 3 T and 7 T before (pre) and after (post) IONPs. All quantitative data represent the mean data of 15 tumors in each group and standard deviations.

## Data Availability

The datasets generated for this study are available from the first author upon request.

## Abbreviations

CCC: Concordance correlation coefficient
CI: Confidence interval
CoV: Coefficients of variation
IONPs: Iron oxide nanoparticles
MRI: Magnetic resonance imaging
TAMs: Tumor-associated macrophages
