# Supplementary figures and images for: Reproducibility and repeatability of quantitative T2 and T2* mapping of osteosarcomas in a mouse model
Source: Eur Radiol Exp. 2024 Jun 14;8:74. doi: 10.1186/s41747-024-00467-9 (PMC11176138; doi:10.1186/s41747-024-00467-9)

## Slide 1
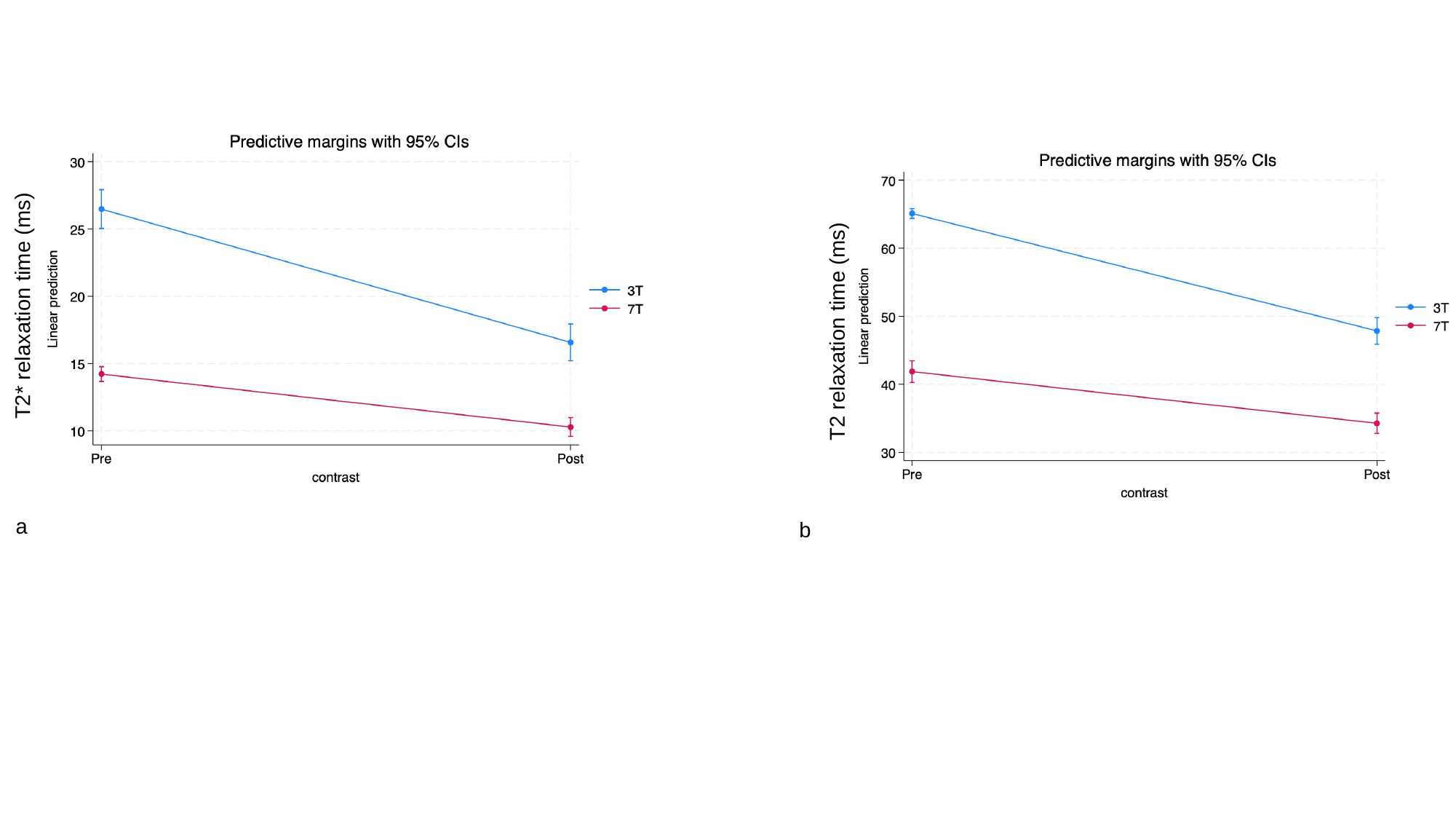

T2* relaxation time (ms)
T2 relaxation time (ms)
a
b

Supplement: Supplementary file 1 — Additional file 1: Supplementary Fig. S1. Tumor T2* and T2 relaxation time measurements before and after iron oxide nanoparticle (IONP) injection: (a) Tumor T2* relaxation time at 3 T and 7 T before (pre) and after (post) IONP injection. (b) T2 relaxation time (ms) at 3 T and 7 T before (pre) and after (post) IONPs. All quantitative data represent the mean data of 15 tumors in each group and standard deviations. [file 41747_2024_467_MOESM1_ESM.pptx]
